# Shoe Sole Tread Designs and Outcomes of Slipping and Falling on Slippery Floor Surfaces

**DOI:** 10.1371/journal.pone.0068989

**Published:** 2013-07-24

**Authors:** Li-Wen Liu, Yung Hui Lee, Chiuhsiang Joe Lin, Kai Way Li, Chih Yong Chen

**Affiliations:** 1 Department of Industrial Management, National Taiwan University of Science and Technology, Taipei, Taiwan, ROC; 2 Department of Industrial Management, Chung Hua University, Hsin-Chu, Taiwan, ROC; 3 Institute of Occupational Safety and Health, Taipei, Taiwan, ROC; McMaster University, Canada

## Abstract

A gait experiment was conducted under two shoe sole and three floor conditions. The shoe soles and floors were characterized by the tread and groove designs on the surface. The coefficients of friction (COF) on the floor in the target area were measured. The subjects were required to walk on a walkway and stepping on a target area covered with glycerol. The motions of the feet of the subjects were captured. Gait parameters were calculated based on the motion data. Among the 240 trials, there were 37 no-slips, 81 microslips, 45 slides, and 77 slips. It was found that the condition with shoe sole and floor had both tread grooves perpendicular to the walking direction had the highest COF, the shortest slip distance, and the lowest percentages of slide and slip. The condition with shoe sole and floor had both tread grooves parallel to the walking direction had the lowest COF and the longest slip distance among all experimental conditions. The Pearson’s correlation coefficients between slip distance and slip velocity, time to foot flat, foot angle, and compensatory step length were 0.82 (*p*<0.0001), 0.33 (*p*<0.0001), −0.54 (*p*<0.0001), and −0.51 (*p*<0.0001), respectively.

## Introduction

Falls are one of the leading causes of death and injury in the workplace [1]. In the UK, one hundred and fifty two workers were killed in 2009 because of falling. This corresponds to a fatality rate of 0.5 per 100,000 workers [2]. In the USA, an average workers’ compensation cost per claim for same-level falls of US$6745 has been reported [3]. In Taiwan, official statistics [4] showed that falling incidences have accounted for more than 15% of all job-related injuries and have been the third most common causes of incidences on workplaces. There were 115 construction worker killed in 2008 because of falling which corresponds to a fatality rate of 1.62 per 100,000 workers [5].

The coefficient of friction (COF) between shoe sole and floor has been adopted to identify the slipperiness level [6]. It is commonly accepted that the lower the COF, the more likely a slip will occur. A measured static COF of 0.5 has been adopted as a safety standard in the USA [7]. Various studies have been conducted to examine factors affecting the COF: the material and surface texture of the footwear and floor, floor contamination condition, inclined angle of the floor surface, and the friction measurement device used [8]–[15].

Measuring friction with mechanical devices on a given floor itself is not enough in understanding the phenomenon of slipping and falling. Supplemental measures of human locomotion when walking are required. Gait parameters associated with slip onset and slip distance has been reported by Perkins [16] and others [17]–[22]. Gait parameters associated with pre-slip were noted for influencing friction demand characteristics at footwear-floor interface 23–26. Gait parameters related to recovery step have also been examined [27]–[30].

Slip-induced instability could result in a fall. Measuring slip distance has been adopted as a direct approach to quantify the risk of slipping and falling [31]. Upon slipping of the foot on the floor, the stability of an individual is jeopardized. Slips that lead to falls are most likely to occur 70–120 ms after the heel touches the ground [16], [8]. In order to avoid loss of balance on a slip, responsive bodily actions are required to retard the forward motion of the slipping foot [6], [17], [27], [32], [,33]. A compensatory step helps to increase bodily stability against loss of balance and to resemble regular gait patterns [29]. A short compensatory step is advantageous in preventing a backward fall.

One of our recent studies [14] examined the effects of shoe sole, floor, contamination, and inclined angle of the floor surface on friction coefficient. It was found that the floors with molded grooves perpendicular to friction measurement direction had the highest friction coefficients than all other floor conditions under both wet and glycerol-contaminated conditions. To better understand the effects of tread grooves on both the shoe sole and floor on the gait parameters associated with slipping of the foot on the floors tested in our previous study, both a friction measurement and a gait experiment was conducted in the current study. Specifically, the purposes of this study were:

- to compare the friction measurements results with the slip outcomes in a gait experiment; and
- to test the hypothesis that the floors with molded grooves perpendicular to walking direction provide the best slip resistance when coupling with the shoe sole with tread grooves in the same direction in a gait; and
- to examine the gait parameters under shoe sole and floor conditions and under levels of slip severity.

## Methods

### Human Subjects

Ten healthy male participants were recruited. All the participants signed informed consent for their participation in the study. The means (±std) of age, stature and weight of the participants were 25.5 (±3.6) years, 167.3 (±3.6) cm, and 61.9 (±9.6) kg, respectively. All the participants had no history of musculoskeletal disease or injury. Employment of human participants was approved by an Institutional Review Board (IRB) of Tri-Service General Hospital.

### Experimental setup

**Figure 1:**
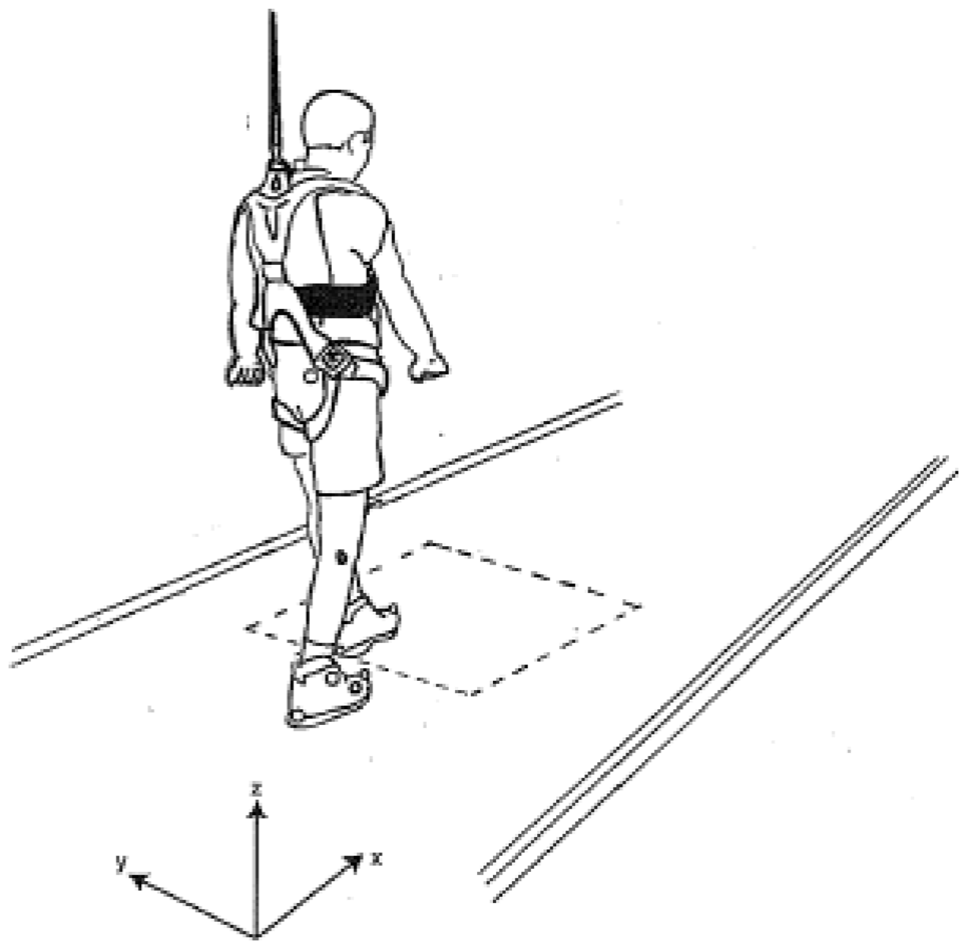
Walkway and target area of the gait experiment.

A 8-m long walkway was constructed for the study. The walkway was covered by a plastic floor pad. A 60 × 60 cm target area was delineated on the floor 3 m from the end of the walkway. A suspending safety harness was installed overhead to prevent the subject from falling (see Fig. 1). Data acquisition was accomplished by using a Vicon® 460 motion analysis system (Oxford Metrics Ltd, Oxford, UK). The sampling rate was 120 Hz. To track the motion of the lower limbs, reflective markers with a diameter of 10 mm were attached on the heel, malleolus, the little toe, first toe, and knee on each leg. The motion data were smoothed using a lowpass-Butterworth-filter with a cut-off frequency of 3 Hz. A LabView® software (National Instruments, TX, USA) was adopted to analyze the spatial coordinates of the markers.

### Floors and footwear conditions

**Figure 2:**
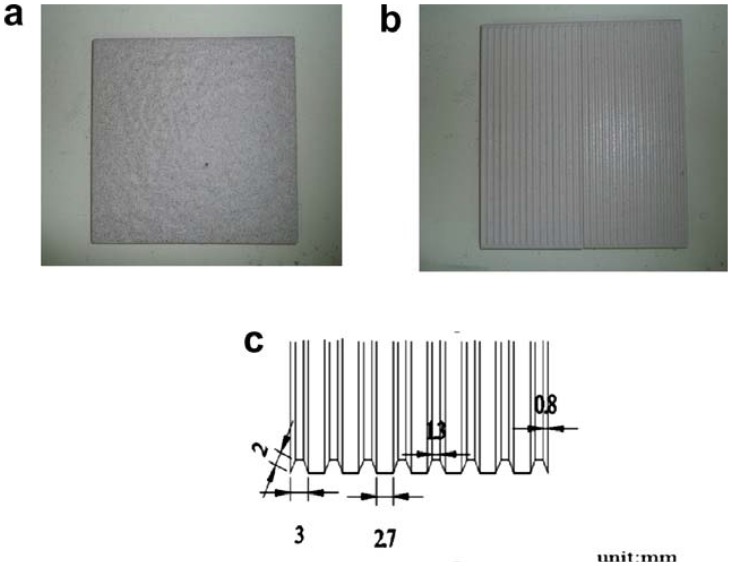
Floor: (a) flat ceramic floor; (b) unglazed ceramic floor with molded trapezoid groove design; (c) dimensions of the groove.

In the target area on the walkway, three floors were prepared. The first one was a flat ceramic floor (FF) surface with R_a,_ also known as the center line average of surface heights (CLA), of 11.1 (±1.0) µm). The second one was an unglazed ceramic floor with molded trapezoid groove design (see Fig. 2). The grooves were perpendicular (FP) to that of the walking direction. The third one was the same unglazed ceramic floor as the second but the grooves were parallel (FL) to that of the walking direction. The R_a_ values at the peaks of those of the second and third floors were both 6.7 (±0.5) µm. In order to generate a slippery floor surface so as to observe slipping of the foot on the floor, an amount (3 ml) of glycerol (weight ratio of 98) was applied evenly on the target area using a paint brush. For the floors with grooves, the glycerol was applied evenly on the top of the peaks. Both the shoe soles and floors were cleaned using a 50% ethanol solution, rinsed with tap water and dried after each test. The same glycerol applying protocol and floor cleaning procedure were adopted for all the subsequent trials.

**Figure 3:**
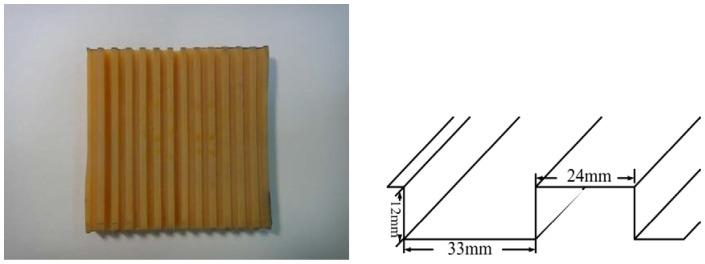
Tread design on the shoe sole.

Shoes with composite rubber shoe sole with various sizes to accommodate all the participants were purchased from a local supplier. The shoe soles had a Shore-A hardness of 69. The shoe sole of the heel had treaded grooves either perpendicular (SP) or parallel (SL) to the walking direction. The dimensions of the tread are shown in Fig. 3.

The COF at the footwear-floor interface in the target area was measured using a Brungraber Mark II slipmeter (Slip Test Inc., Spring Lake, NJ, USA). The footwear, floor, and surface conditions exactly the same as those of the gait experiment were tested. A sample of 40 for each footwear and floor conditions were recorded. The standard test method for the BM II proposed by the American Society of Testing and Materials [34] was adopted. The friction measurement protocol followed those in Li et al. [35].

### Tempo-spatial variables

**Figure 4:**
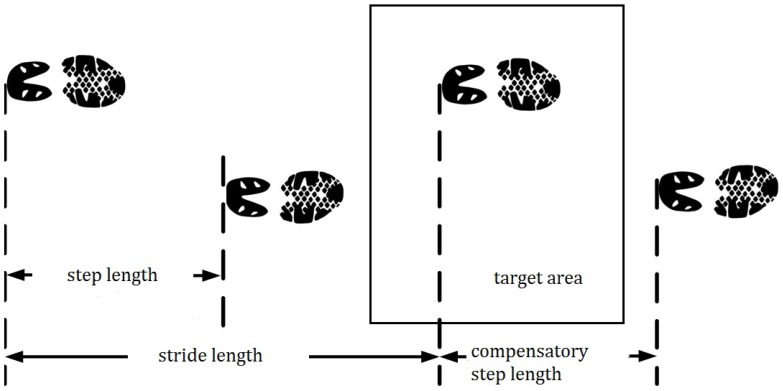
Step length, stride length, and compensatory step length.

Tempo-spatial variables of slippage were selected to reflect the motion patterns of the foot during walking. The spatial coordinates of the markers on the lower limbs were used to calculate the gait variables. The gait cycle time was normalized to stance duration, with 0% being heel contact and 100% representing toe-off the target zone. A heel strike was identified when the vertical coordinate of the heel marker reach a minimum. The end of a slip was identified when the velocity of the heel marker reached 0. The tempo-spatial variables are defined below:

Step length: distance between the heel markers during two consecutive heel strikes before stepping on the target zone (see Fig. 4);

Stride length: The stride length is the distance between two successive placements of the same foot on the floor (see Fig. 4);

Walking velocity: the stride length divided by the gait cycle time;

Foot angle: the angle subtended by the heel and floor in the sagittal plane at the time of heel strike;

Time to foot flat: the period between heel strike and foot flat;

Slip distance: The traveling distance of the heel marker in the target zone from heel strike to slip stop;

Slip velocity: slip distance divided by the slip time;

Compensatory step length: the step length starting with left foot on the target area (see Fig. 4);

### Experiment procedure

Each participant had, at least, fifteen minutes to acclimatize to the experimental setup before data collection. In this period, the participant put on the lab shoes, wore the safety harness and walked over the dry walkway. The participants were instructed to walk as naturally as possible with a cadence of 120 steps/min by metronome and looking at the frontto minimize visual cues as to the floor conditions. The participant was encouraged to step on the target area. A stopwatch was used to record the time and to provide feedback for the participant in order to maintain consistent walking speed. In case when the subject lost cadence or failed to stepping on the target zone, the trial would be abandoned and he will be requested to walk again.

In the experiment, the order of different shoe-floor conditions was randomly assigned. The participant then walked on the walkway toward the target zone just as the same way he had done in the practice prior the test. Glycerol was applied on the target area before the experiment started. The motion data of the lower limbs of the participant were recorded using the motion tracking system. A trial stopped after the participant passed the target area.

### Experiment design and Data analysis

An experiment with a randomized complete block design was adopted. Each participant was regarded as a block. The six experimental conditions were randomized within each participant. Four trials were included for each treatment in each block and were performed in succession. There were a total of 240 trials (10 subjects×3 floor conditions×2 shoe conditions×4 trials). Analysis of variance (ANOVA) with repeated measures was used to test the effects of shoe and floor patterns and the interactions between these the factors. Duncan’s multiple range tests were conducted for post hoc comparisons. The Pearson’s correlation coefficients between slip distance and other gait parameters were calculated. Statistical analyses were performed using the SPSS® 17.0 (SPSS® Inc., Chicago, Illinois).

## Results

### COF at the shoe-floor interface

Table 1 shows the COF values measured under all the six shoe-floor conditions. The COF in FP-SP condition (0.56) was the highest among all experimental conditions. All other COFs, ranged from 0.11 to 0.16, were lower than 0.5 which has been adopted as a safety standard in the USA [7].

**Table 1:** COF values under shoe-floor conditions.

Floor	Shoe sole	mean	std
FP	SP	0.56	0.02
FL	SP	0.13	0.01
FP	SL	0.16	0.01
FL	SL	0.11	0.01
FF	SP	0.15	0.01
FF	SL	0.12	0.01

### ANOVA results of the tempo-spatial variables

One two-way ANOVA was performed for each of the tempo-spatial variables. Duncan’s multiple range tests were performed if the main effects of shoe sole and floor reached statistically significant level (α = 0.05). Letters of homogeneous subsets within the significant factor were, then, marked. Results of the tempo-spatial variables are shown in Table 2. The means and standard deviations for the conditions in each factor (shoe and floor surfaces) were calculated across the other factor. With a controlled cadence of 120 steps/min, the step length (ranged 69.0±6.5 cm to 71.8±4.3 cm), stride length (ranged 128.8±10.3 cm to 133.9±8.7 cm) and walking velocity (ranged 1.76±0.22 m/s to 1.90±0.35 m/s) were relatively consistent across the experimental conditions.

**Table 2:** Tempo-spatial variables under shoe, floor and shoe × floor conditions.

	STEL (cm)	STRL (cm)	WV (m/s)	FA (°)	SDI (cm)	SV (m/s)	TFF (s)	CSL (cm)
Shoe								
SP	70.1(6.1)	130.2(10.8)	1.85(0.29)	16.4(7.6)	7.4^***^(10.5)	0.73*(0.37)	0.268*(0.074)	65.0(8.5)
SL	70.7(6.3)	131.5(9.9)	1.83(0.25)	16.6(7.9)	9.9^***^(10.5)	0.79*(0.31)	0.285*(0.071)	64.2(8.8)
Floor								
FP	69.6(5.8)	131.2* ^ab^(10.1)	1.87^**a^(0.28)	19.1^***a^(8.3)	3.9^***a^(6.3)	0.60^***a^(0.28)	0.266* ^a^(0.074)	66.9^***a^(7.5)
FL	71.2(5.2)	132.3* ^b^(10.7)	1.87^**a^(0.29)	15.6^***b^(7.8)	10.5^***b^(10.8)	0.82^***b^(0.31)	0.274* ^ab^(0.079)	62.8^***b^(9.1)
FF	70.5(7.3)	129.1* ^a^(10.2)	1.78^**b^(0.24)	14.7^***b^(6.4)	11.4^***b^(12.1)	0.86^***b^(0.38)	0.290* ^b^(0.062)	64.1^***b^(8.9)
Floor×Shoe								
FP-SP	69.0(6.5)	130.9(9.8)	1.88(0.27)	19.0(8.1)	1.6(1.3)	0.51* ^a^(0.19)	0.251(0.064)	67.4(6.2)
FL-SP	70.7(5.9)	130.8(12.3)	1.90(0.35)	15.6(7.4)	10.0(12.5)	0.81* ^bc^(0.35)	0.266(0.087)	62.5(9.6)
FP-SL	70.1(5.1)	131.4(10.6)	1.85(0.28)	19.3(8.6)	6.3(8.2)	0.69* ^b^(0.32)	0.281(0.080)	66.4(8.7)
FL-SL	71.8(4.3)	133.9(8.7)	1.85(0.22)	15.6(8.3)	10.9(9.1)	0.83* ^bc^(0.26)	0.281(0.071)	63.2(8.6)
FF-SL	70.3(8.7)	129.3(10.2)	1.80(0.26)	14.9(6.2)	12.5(12.9)	0.85* ^b^(0.34)	0.292(0.061)	63.0(9.0)
FF-SP	70.7(5.9)	128.8(10.3)	1.76(0.22)	14.5(6.7)	10.4(11.2)	0.87* ^b^(0.42)	0.288(0.064)	65.2(8.7)

* *p*<0.05;^ **^ *p*<0.01; ^***^ *p*<0.001; a, b, c: homogeneous subsets by Duncan analysis with α = 0.05; FF = flat floor; FP = floor with groove-perpendicular; FL = floor with groove-parallel; SP = shoe-groove-perpendicular; SL =  shoe-groove-parallel ; STEL: step length; STRL: stride length; WV: walking velocity; FA: foot angle; SDI: slip distance; SV: slip velocity; TFF: time to foot flat; CSL: compensatory step length.

The ANOVA results showed significant effects for shoe sole (*p* = 0.001) on slip distance. The trials with SP conditions had significant (*p*<0.05) lower slip distance (7.4±10.5 cm) than those of the SL conditions (9.9±10.5 cm). The SP conditions also showed significant (*p*<0.05) lower time to foot flat (0.268±0.074 m/s) than those of the SL conditions (0.285±0.071 m/s).

The ANOVA results also showed significant floor effects on all the eight tempo-spatial variables except step length in Table 2. The FP condition showed significant (*p*<0.05) lower slip distance (3.9±.6.3 cm) than those of the FL (10.5±10.8 cm) and FF (11.4±12.1 cm) conditions. Similar results were also observed in foot angle, slip velocity, and compensatory slip length.

Significant (*p*<0.05) shoe-floor interaction effects on both slip distance and slip velocity indicated that the effects of floor conditions differ in different shoe conditions. The FP-SP condition had significantly the shortest slip distance (1.6±1.3 cm) than all other shoe-floor conditions. The FF-SL condition had the longest slip distance (12.5±12.9 cm) which was significantly higher than those of the FP-SP and FP-SL conditions.

### Outcomes of trials

A total of 240 trials were tested in this study. The outcome of every trial was identified as one of the four categories: no-slip, microslip, slide, and slip based on the slip distance. A trial with a slip distance of 1 cm or less was termed a no-slip. Microslips were those with a slip distances greater than 1 cm and less 3 cm [31]. Slides were the trials with slip distances ranged 3 to 10 cm. The trials with slip distances of 10 cm or more were termed slip [36]. All the slip trials could end up with falls without the protection of the safety harness. Table 3 shows the percentages of each category under the experimental conditions. For the most slip resistant condition (FP-SP, COF = 0.56), the percentages of no-slip, microslip, slide, and slip were 45%, 32.5%, 22.5%, and 0%, respectively. For the most slippery condition (FL-SL, COF = 0.11), the percentages of no-slip, microslip, slide, and slip were 5%, 22.5%, 25%, and 47.5%, respectively. However, the FF-SL condition, which had slightly higher COF (0.12) than the FL-SL condition, showed the highest percentage of slip (50%). The outcomes of the trials seemed to be consistent with those of the friction measurement results.

**Table 3:** Outcomes of the trials (%).

floor	shoe sole	no-slip	microslip	slide	slip
FP	SP	45.0	32.5	22.5	0.0
FL	SP	7.5	52.5	5.0	35.0
FP	SL	12.5	42.5	22.5	22.5
FL	SL	5.0	22.5	25.0	47.5
FF	SP	15.0	25.0	22.5	37.5
FF	SL	7.5	27.5	15.0	50.0

Among the 240 trials, there were 37 no-slips, 81 microslips, 45 slides, and 77 slips. Table 4 shows the tempo-spatial variables in different experimental outcomes. One ANOVA was performed for each of the tempo-spatial variables in Table 4 using the slip category as the independent variable. Duncan’s multiple range tests were conducted and letters of homogeneous subsets were marked if the slip category was statistically significant (α = 0.05) on the dependent variable. The slip distance for no-slip, microslip, slide, and slip were 0.51 (±0.31) cm, 1.75 (±0.54) cm, 5.28 (±0.31) cm, and 21.64 (±9.30) cm, respectively.

**Table 4:** Tempo-spatial variables in different slip categories.

	Step length* (cm)	Stride length* (cm)	Walk Velocity* (m/s)	Foot angle* (°)	slip distance* (cm)	slip velocity* (m/s)	Time to foot flat* (s)	compensatory step length* (cm)
All trials	70.4(6.2)	130.9 (10.4)	1.84 (0.27)	16.5 (7.8)	8.61(10.55)	0.76(0.34)	0.28(0.07)	64.6(8.7)
No-slip	69.8^ab^(6.3)	135.0^ a^ (7.7)	1.94^ a^ (0.19)	21.2^ a^ (6.4)	0.51^ a^ (0.31)	0.43^ a^ (0.13)	0.24^ a^ (0.05)	68.8 (7.0)
Microslip	69.1^ a^ (6.5)	132.4^ a^ (9.3)	1.83^b^ (0.27)	21.0^ a^ (5.2)	1.75^ a^ (0.54)	0.60^ b^ (0.14)	0.27^ b^ (0.05)	67.1 (8.1)
Slide	70.8^ ab^ (3.1)	131.7^ a^ (3.1)	1.85^ ab^ (3.05)	15.0^ b^ (8.7)	5.28^ b^ (0.31)	0.75^ c^ (3.05)	0.28^ bc^ (0.07)	67.1 (8.1)
slip	71.8^ b^ (5.4)	126.8^ b^ (10.5)	1.79^ b^ (0.29)	10.3 ^c^ (5.1)	21.64^b^ (9.30)	1.09^ d^ (0.36)	0.30^ c^ (0.09)	65.7 (7.0)

* *p*<0.0001; a, b, c: homogeneous subsets by Duncan analysis with α = 0.05;

The Pearson’s correlation coefficients between slip distance and slip velocity, time to foot flat, foot angle, and compensatory step length were 0.82 (*p*<0.0001), 0.33 (*p*<0.0001), −0.54 (*p*<0.0001), and −0.51 (*p*<0.0001), respectively.

## Discussion

Humans usually adopt preventive gait patterns to reduce slip potential when anticipating slippery surface, even when instructed to walk as naturally as possible [20]. Visual perception is one of the important factors affecting the gait pattern of a walker [37]. Glycerol was applied in the target area for all the trials. The participants could not see the tread pattern on the shoe soles. The only visual cue was whether the floor was flat or had grooves either in perpendicular or parallel to the walking direction. Effects of visual perception on the gait patterns among the experimental conditions were believed to be minor and might, therefore, be neglected. This was supported by the facts that the gait patterns before stepping on the target area including step length, stride length, and walking velocity were consistent across the experimental conditions. Variations for gait parameters measured on or after heel contacted the floor, including the foot angle, slip distance, slip velocity, time to foot flat, and compensatory step length, could solely be attributed to the effects of the shoe sole-floor interface.

The foot angle has been one of the important gait parameters in examining heel dynamics of slipping. The foot angles, ranged 14.5° to 19.3°, in the six experimental conditions (see Table 2) were lower than those reported in the literature [17], [20], [21]. It was found that the foot angle for the slip trials (10.3°, see Table 4) was especially low. The foot angles for the no-slip (21.2°) and microslip (21.0°) trials, however, were similar to those of Strandberg and Lanshammar [17] and Cham and Redfern [20] but were still lower than those in McGorry et al. [21] (25.1° and 25.3 for non-slip & slip conditions, respectively). The reason for the low foot angle in the slip trials might be that the participants took preventive gait strategy by reducing their foot angles [20], [25] immediately before they stepped on the target area when they anticipated slippery condition. How such anticipation was built up was still not clear as the conditions before stepping on the target area was the same for all the experimental conditions and the participants did not receive proprioceptive feedback before they slipped.

The literature has suggested that a fall could occur if the peak slip velocity of the heel is more than 0.5 m/s [16], [17]. All the slip velocities in the six experimental conditions (see table 2) exceeded this limit. This seemed to be contradictory with that suggested by Perkins [16] and Strandberg and Lanshammar [17]. However, the results of the current study indicated that the slip distance was highly correlated with the slip velocity (*r* = 0.82; *p*<0.0001). In addition, only the slip velocity of the no-slip condition in Table 4 was below 0.5 m/sec. The slip velocities for microslip and slide were 0.6 and 0.75 m/sec, respectively. The slip velocity for slip was even as high as 1.09 m/sec. This was partially consistent with those of McGorry et al. [21] where they reported instantaneous heel velocities of 1.0 and 1.1 m/sec for the non-slip and slip conditions, respectively. The results of Cham and Redfern [20] also showed that the slip velocities associated with their fall trials were greater than or equal to 0.8 m/sec. Brady et al. [32] even suggested that individuals were able to avoid falls for slips with peak slip velocities far exceeding 1.0 m/sec.

The compensatory step length has been one of the gait parameters associated with recovery attempts when slipping of the foot was detected. The gait was disturbed when the leading foot was slipping and early landing of the lagged foot was required to achieve dual support from both feet. The compensatory step would increase stability against further backward balance loss and resemble the regular gait pattern [29]. A short compensatory step length was associated with long slip distance which was likely to result in a fall. This was supported by the negative correlation between the slip distance and the compensatory step length (*r* = −0.51; *p*<0.0001).

One of the hypotheses of the study was that the FP-SP combination provides the best slip resistant effect because of the mechanical interlocking effect among all shoe and floor conditions. This hypothesis was supported by the results of both the friction measurements and gait experiment. The FP-SP conditions had significantly (*p*<0.05) the highest COF (0.56) among all shoe-floor conditions. This was consistent with the findings in the literature [14].

The results of the friction measurements were consistent with those of the gait experiment. There was no slip observed in the most slip resistant condition (FP-SP). Most of the trials (77.5%) under this condition were either no-slip or microslip. The slip distance under this condition was also significantly (*p*<0.05) shorter than that of any other experimental conditions. It was obvious that treads on both the shoe sole and floor interlock especially when both were perpendicular to the walking direction. Forward movement of the sole was obstructed when the tread on the sole was “trapped” in the grooves on the floor. The FL-SL, on the other hand, showed the lowest COF (0.11) and poor gait experimental outcomes (72.5% of the trials were either slide or slip). A combination of tread grooves both on shoe sole and the floor parallel to the walking direction provided sliding trail which facilitate forward movement of the shoe on the floor. Tread grooves both on shoe sole and on the floor parallel to the walking direction should, therefore, be avoid. This was consistent with the findings in Li and Chen [12].

The limitation of the study was that, like many other gait experiments, the walkway environment was artificial and anticipation effect was a constant factor across all trials [20], [24], [25]. In addition, the controlled cadence at 120 steps/min, which resulted in walking velocities ranging from 1.76 to 1.9 m/sec, was deliberately selected. Even though the walking velocities were consistent with those in the literature [21], those velocities may not reflect the real conditions in people’s daily life especially when they anticipate slippery floor surfaces. Finally, only the SP-FP conditions had COF value over 0.5. The COF values of the other five shoe-floor conditions were in a small range (0.11∼0.16). This could result in limited sensitivity of the data in explaining the gait patterns of the participants.

## Conclusions

A gait experiment was conducted under two shoe sole and three floor conditions. The participants were required to walk on a walkway and stepping on a target area. Both the shoe sole and floor had tread grooves perpendicular to the walking direction provided the best interlocking effects which resulted in a COF of 0.56 even the floor was glycerol-contaminated. This result was consistent with the findings for the slip distance in the gait experiment under the same shoe-floor condition. The gait parameters of our experiment are helpful in understanding the foot dynamic on floors with molded groove designs.
